# Severe QT interval prolongation associated with moxifloxacin: a case report

**DOI:** 10.1186/1757-1626-1-409

**Published:** 2008-12-19

**Authors:** Tetsuro Koide, Masato Shiba, Katsuhiro Tanaka, Masatoshi Muramatsu, Satoshi Ishida, Yoshihiro Kondo, Keiko Watanabe

**Affiliations:** 1 Department of Pharmacy, Kuwana Municipal Hospital, 430 Kitabessyo, Kuwana 511-0819, Japan; 2 Department of Neurosurgery, Kuwana Municipal Hospital, 430 Kitabessyo, Kuwana 511-0819, Japan; 3 Department of Internal Medicine, Kuwana Municipal Hospital, 430 Kitabessyo, Kuwana 511-0819, Japan

## Abstract

**Introduction:**

The QT interval prolongation is an adverse effect associated with moxifloxacin. This adverse effect can lead to potentially life-threatening arrhythmias such as Torsades de pointes. We describe a case of severe QT interval prolongation associated with moxifloxacin which may cause the development of Torsades de pointes. There have been no reported case of severe corrected QT interval prolongation caused by moxifloxacin in the patient of normal heart rate.

**Case presentation:**

In an 85-year-old Japanese woman, oral moxifloxacin 400 mg daily was initiated for the forearm cellulitis. On the sixth day of oral moxifloxacin administration, monitor electrocardiogram showed prolongation of the corrected QT interval to 523 ms at a rate of 40 beats/min. Electrocardiogram before moxifloxacin therapy showed the corrected QT interval to 460 ms at a rate of 72 beats/min. On the sixth day after moxifloxacin discontinuance, monitor electrocardiogram showed the corrected QT interval to 432 ms at a rate of 70 beats/min.

**Conclusion:**

This case suggests that electrocardiogram monitoring during moxifloxacin therapy may be necessary in the patients even if they do not have high risk factors for QT interval prolongation.

## Introduction

Fluoroquinolones are clinically important antibiotic drugs. Although they are generally well tolerated, with safety profiles similar to those of other antimicrobial agents, they may sometimes result in significant adverse reactions [1]. The QT interval prolongation is an adverse effect associated with fluoroquinolones. It can lead to potentially life-threatening arrhythmias such as Torsades de pointes (TdP). Moxifloxacin is one of the third-generation fluoroquinolones with a broad spectrum of activity, including gram-negative, gram-positive bacteria, anaerobes and atypical pneumonia agents [1]. TdP associated with moxifloxacin have been reported in the extreme bradycardia patients who need cardiac pacemaker implantation [2,3]. We describe a case of severe QT interval prolongation associated with moxifloxacin which may cause the development of TdP. There have been no reported case of severe corrected QT (QTc) interval prolongation caused by moxifloxacin in the patient of normal heart rate.

## Case presentation

**Figure 1:**
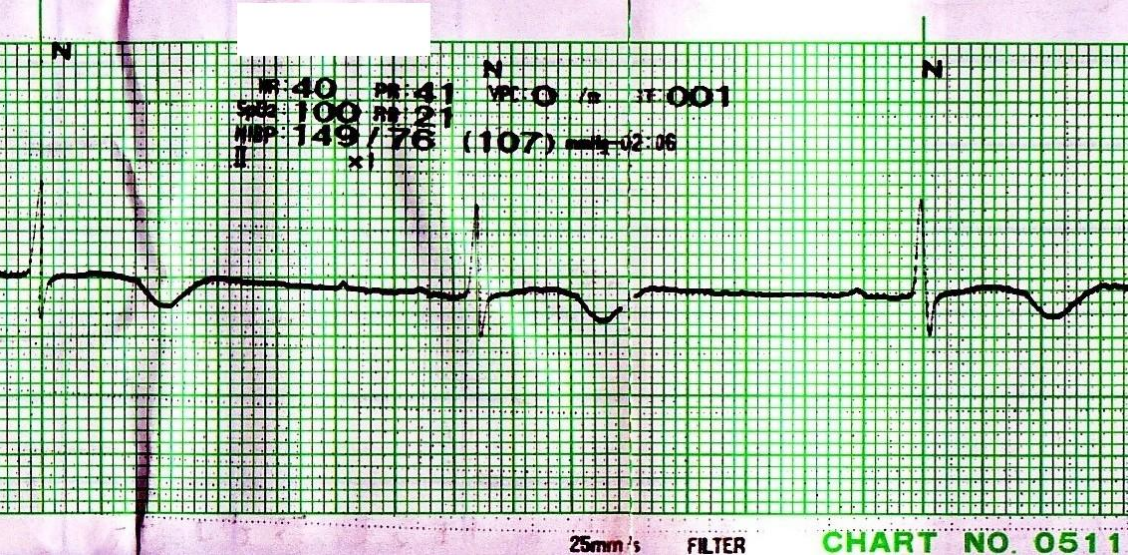
**Monitor ECG on the sixth day of moxifloxacin administration; Monitor ECG on the sixth day of oral moxifloxacin therapy showed prolongation of the QTc interval to 523 ms with a rate of 40 beats/min**.

**Figure 2:**
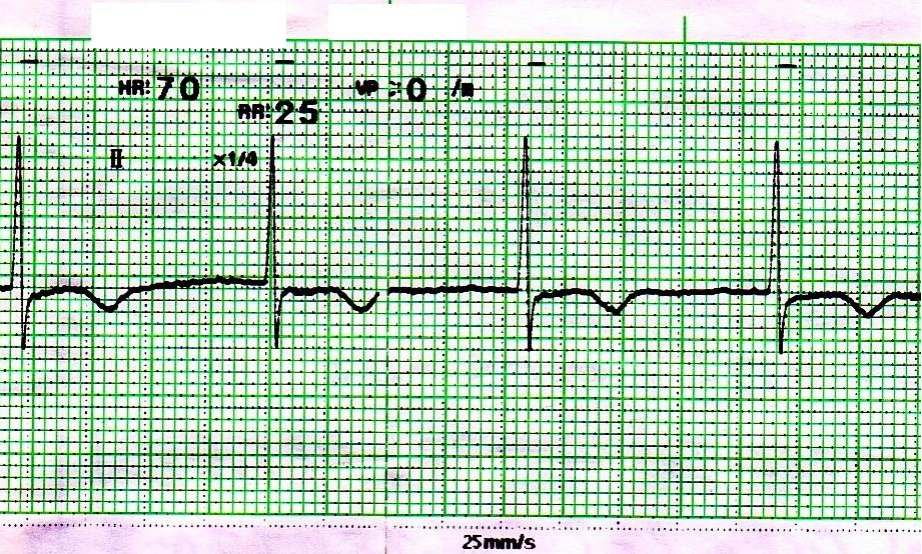
**Monitor ECG on the sixth day after moxifloxacin discontinuance; Monitor ECG on the sixth day after moxifloxacin discontinuance showed the QTc interval to 432 ms with at a rate of 70 beats/min**.

An 85-year-old unconscious Japanese woman with hypertension, dementia was admitted to the neurosurgery department of our hospital. Her height was about 140 cm and she weighed 35 kg. On admission, her head Computed Tomography (CT) illustrated the fourth ventricle hemorrhage with hydrocephalus. Admission electrocardiogram (ECG) showed the QT interval to 420 ms with a sinus rhythm at a rate of 72 beats/min. According to Bazett's formula, the QTc interval was 460 ms. Medications she was using just before admission included slow-release nifedipine 10 mg twice daily and famotidine 10 mg twice daily. After her admission, these medications were stopped because of her unconsciousness. Clinical laboratory findings at the time of admission were as follows; serum aspartate-amino transferase level was 22 IU/L, serum alanine transaminase level was 9 IU/L, serum total bilirubin level was 0.7 mg/dl, serum potassium level was 4.0 mEq/L, serum urea nitrogen level was 25.9 mg/dl and serum creatinine level was 0.6 mg/dl. Thus, renal dysfunction, hepatic dysfunction and hypokalemia were not observed on admission. Other biochemical tests were normal. On the fifth day after admission, the ventriculo-peritoneal shunt was performed for hydrocephalus. As the result, her consciousness was improved remarkably, therefore slow-release nifedipine and famotidine were restarted on the seventh day after admission. On the twenty-first day after hospitalization, the right forearm pain and swelling were developed, so oral moxifloxacin 400 mg daily was initiated for the right forearm cellulitis. In a few days, the right forearm pain and swelling were reduced. But on the sixth day of moxifloxacin administration, her pulse rate indicated 40 beats/min that was bradycardia, so monitor ECG was performed and it showed prolongation of the QT interval to 640 ms. Using Bazett's formula, the QTc interval was 523 ms (Figure 1). When the severe QTc interval prolongation was developed, the serum electrolytes, renal function and hepatic function were normal. We assumed that the cause of severe QTc interval prolongation was moxifloxacin, because the severe QTc interval prolongation was observed after moxifloxacin therapy. Therefore, moxifloxacin was discontinued. Furthermore, because the cases of QT interval prolongation associated with famotidine have been reported [4], famotidine was also stopped. On the sixth day after moxifloxacin discontinuance, monitor ECG showed the QT interval to 400 ms with a sinus rhythm at a rate of 70 beats/min. According to Bazett's formula, the QTc interval was 432 ms that was improved to normal range (Figure 2).

## Discussion

Moxifloxacin, like other drugs that cause an acquired long QT syndrome, prolongs the QT intrerval by blocking the rapid component of the delayed rectifier potassium current (IKr) in the heart [5]. Inhibition of IKr delays cardiac repolarization by blocking potassium ions flow out of myocytes. The potency of IKr blockade and QT prolongation by moxifloxacin varies dose-dependently. Risk factors of the QT interval prolongation include female sex, advanced age, history of long QT syndrome, organic heart disease, bradycardia, electrolyte disturbances (particularly hypokalemia, hypomagnesemia, hypocalcemia), renal dysfunction, hepatic dysfunction, overdosing and co-administration of drugs that prolong the QT interval [6]. Females generally have QTc interval about 13 ms longer than males. The QTc interval of advanced age prolongs approximately 6 ms compared with that of young age [7].

According to the Naranjo adverse drug reaction probability scale, the severe QTc interval prolongation was probably related to moxifloxacin administration in this case [8]. In our patient, the QTc interval was prolonged to 523 ms, which indicated the increase of 63 ms from her baseline range. The QTc interval over 500 ms or increase from baseline of more than 60 ms is a cause of TdP, according to the Food and Drug Administration and the Committee for Proprietary Medicinal Products [9]. Therefore, TdP might have developed if moxifloxacin were not stopped in our patient.

Risk factors such as organic heart failure, electrolyte abnormalities, renal dysfunction and hepatic dysfunction were not observed in our case. When the severe QTc interval prolongation was developed, famotidine was also stopped with moxifloxacin, because the cases of QT interval prolongation associated with famotidine have been reported [4]. Six days after moxifloxacin and famotidine cessation, the QTc interval was improved to normal range rather than the baseline of our patient. Her baseline QTc interval might have been slightly prolonged by famotidine. Furthermore, famotidine might have assisted in the development of severe QTc prolongation associated with moxifloxacin.

There have been two reported cases of TdP associated with moxifloxacin [2,3]. These two patients had multiple risk factors for QTc interval prolongation (female sex, advanced age, extreme bradycardia, slight baseline QTc interval prolongation). Particularly, they had extreme bradycardia that needed the implantation of cardiac pacemaker. At lower heart rates, less potassium moves out of the cells, because the cardac repolarizations are fewer, reducing the extracellular potassium concentration. Since the potency of IKr inhibition is reversely related to the extracellular potassium concentration, this reduction enhances the degree of IKr blockade [10]. Furthermore, IKr inhibitor such as moxifloxacin enhances the magnitude of IKr inhibition additively. Therefore it seems that the severe bradycardia assisted the development of TdP associated with these two moxifloxacin therapy. Our patient also had some risk factors for QTc interval prolongation (female sex, advanced age, slight baseline QTc interval prolongation, co-administration of drugs that may prolong the QT interval), however bradycardia was not observed at the time of admission. On the sixth day of moxifloxacin administration, the severe QTc prolongation with bradycardia was observed for the first time.

## Conclusion

We describe the severe QT interval prolongation caused by moxifloxacin in the patient of normal heart rate. This case suggests that ECG monitoring during moxifloxacin therapy is necessary in the patients having some risk factors for QTc interval prolongation (female sex, advanced age, slight baseline QTc interval prolongation, co-administration of drugs that may prolong the QT interval).

## Abbreviations

TdP: Torsades de pointes; QTc: corrected QT; ECG: electrocardiogram; CT: Computed Tomography; IKr: the rapid component of the delayed rectifier potassium current.

## Consent

Written informed consent was obtained from the patient's next-of-kin for publication of this case report and accompanying images. A copy of the written consent is available for review by the Editor-in-Chief of this journal.

## Competing interests

The authors declare that they have no competing interests.

## Authors' contributions

TK was involved in the patient's monitoring, pharmaceutical care, literature review and manuscript preparation, editing and submission. MS and KT were involved in the patient's evaluation and clinical care. MM and SI were involved in the patient's evaluation and clinical care and they reviewed the manuscript. YK was involved in the patient's monitoring and pharmaceutical care. KW reviewed the manuscript.
